# Whole Blood Transcriptome Analysis in Congenital Anemia Patients

**DOI:** 10.3390/ijms252111706

**Published:** 2024-10-31

**Authors:** Maria Sanchez-Villalobos, Eulalia Campos Baños, Elena Martínez-Balsalobre, Veronica Navarro-Ramirez, María Asunción Beltrán Videla, Miriam Pinilla, Encarna Guillén-Navarro, Eduardo Salido-Fierrez, Ana Belén Pérez-Oliva

**Affiliations:** 1 Biomedical Research Institute of Murcia (IMIB-Pascual Parrilla), 30120 Murcia, Spainlalycampos19@gmail.com (E.C.B.); asuncionbeltranvi@gmail.com (M.A.B.V.);; 2 Hematology Service, Virgen de la Arrixaca University Hospital, 30120 Murcia, Spain; 3 Medical Genetics Section and Pediatrics Service, Virgen de la Arrixaca University Hospital, University of Murcia, 30120 Murcia, Spain; 4 CIBERER-ISCIII, 28029 Madrid, Spain

**Keywords:** blood disorders, transcriptomic analysis, congenital sideroblastic anemia (CSA), β-thalassemia, sickle cell disease (SCD)

## Abstract

Congenital anemias include a broad range of disorders marked by inherent abnormalities in red blood cells. These abnormalities include enzymatic, membrane, and congenital defects in erythropoiesis, as well as hemoglobinopathies such as sickle cell disease and thalassemia. These conditions range in presentation from asymptomatic cases to those requiring frequent blood transfusions, exhibiting phenotypic heterogeneity and different degrees of severity. Despite understanding their different etiologies, all of them have a common pathophysiological origin with congenital defects of erythropoiesis. We can find different types, from congenital sideroblastic anemia (CSA), which is a bone marrow failure anemia, to hemoglobinopathies as sickle cell disease and thalassemia, with a higher prevalence and clinical impact. Recent efforts have focused on understanding erythropoiesis dysfunction in these anemias but, so far, deep gene sequencing analysis comparing all of them has not been performed. Our study used Quant 3′ mRNA-Sequencing to compare transcriptomic profiles of four sickle cell disease patients, ten thalassemia patients, and one rare case of SLC25A38 CSA. Our results showed clear differentiated gene map expressions in all of them with respect to healthy controls. Our study reveals that genes related to metabolic processes, membrane genes, and erythropoiesis are upregulated with respect to healthy controls in all pathologies studied except in the SLC25A38 CSA patient, who shows a unique gene expression pattern compared to the rest of the congenital anemias studied. Our analysis is the first that compares gene expression patterns across different congenital anemias to provide a broad spectrum of genes that could have clinical relevance in these pathologies.

## 1. Introduction

Congenital anemias encompass a wide spectrum of diseases which can be classified into two main groups. On the one hand, there are hereditary hemolytic anemias, whose origin is an intrinsic alteration of the RBC: enzymatic deficiencies (the most representative is pyruvate kinase deficiency, PKD); structural defects of the red blood cell membrane (the most representative is hereditary spherocytosis); and hemoglobin disorders (hemoglobinopathies and thalassemia syndromes). On the other hand, anemias secondary to congenital defects of erythropoiesis, such as dyserythropoietic anemias and congenital sideroblastic anemias, are mainly characterized by bone marrow failure [1].

The various congenital anemia syndromes can have similar or highly variable clinical and laboratory presentations, from asymptomatic patients whose diagnosis goes unnoticed (e.g., spherocytosis, which has less need for treatment) to patients with high transfusion requirements.

Despite a good understanding of the etiology of these inherited disorders, there are still many unknown factors associated with their phenotypic heterogeneity and there is a clinical need to better understand the gene signature expression of these patients. Congenital anemias are multisystemic diseases in which patients diagnosed with the same mutations may present a variable range of anemia severity [2]. However, the pathophysiology is different depending on whether it is a defect in the red blood cell itself or a defect in erythropoiesis that causes ineffective erythropoiesis. In our work, we divide congenital hemolytic anemias into two large groups: those in which the origin is in an intrinsic defect of the red blood cell, that is, of its components (membranopathies, enzymopathies, and hemoglobinopathies), and those in which the primary defect is due to a bone marrow failure (such as congenital dyserythropoietic anemia (CDA) and CSA).

Significant efforts have been made in recent years to understand the pathophysiology of dysfunctional erythropoiesis in congenital anemias [3]. While several studies have analyzed peripheral blood transcriptome profiles in sickle cell disease (SCD) patients [4,5,6], data on other congenital anemias remain scarce, with no comparative studies available. In this study, we used Quant 3′ mRNA-Sequencing, a method to analyze gene expression comprehensively, especially in low-quality samples, such as in congenital anemia patient samples. In our study, we performed a gene profile expression analysis in patients diagnosed with the following: congenital hemolytic anemias (SCD); transfusion-dependent thalassemia (TDT); non-transfusion-dependent thalassemia (NTDT); and bone marrow failure represented by a patient with a disorder of SLC25A38 CSA. Moreover, a patient included in the TDT group has heterozygous β-thalassemia and PKD. The genes selected for study are representative of several signaling pathways important in erythropoiesis, iron metabolism, glycolysis, oxidative metabolism, and red blood cell membrane proteins. All these genes work together in a complex network to ensure the proper development and function of red blood cells, and this is the reason why they were selected in our study.

Despite the heterogeneity present in congenial anemias, there is a duality of dyserythropoiesis/ineffective erythropoiesis vs. hemolysis in all of them and at the pathophysiological level. Our findings reveal a distinct differential expression profile in all patients studied compared to the healthy controls. Moreover, we observed a unique gene profile in the SLC25A38 patient with respect to other congenital anemia groups. Our study provides, for the first time, a unique gene map profile that plays a role in understanding the pathophysiology of congenital anemia, which has potentially useful implications for further elucidating the pathophysiology and the clinical variability of the different congenital anemias, as well as for aiding the diagnosis of unclear cases.

## 2. Results

### 2.1. Gene Profile Expression of Different Congenital Anemias with Respect to Healthy Donors

**Figure 1:**
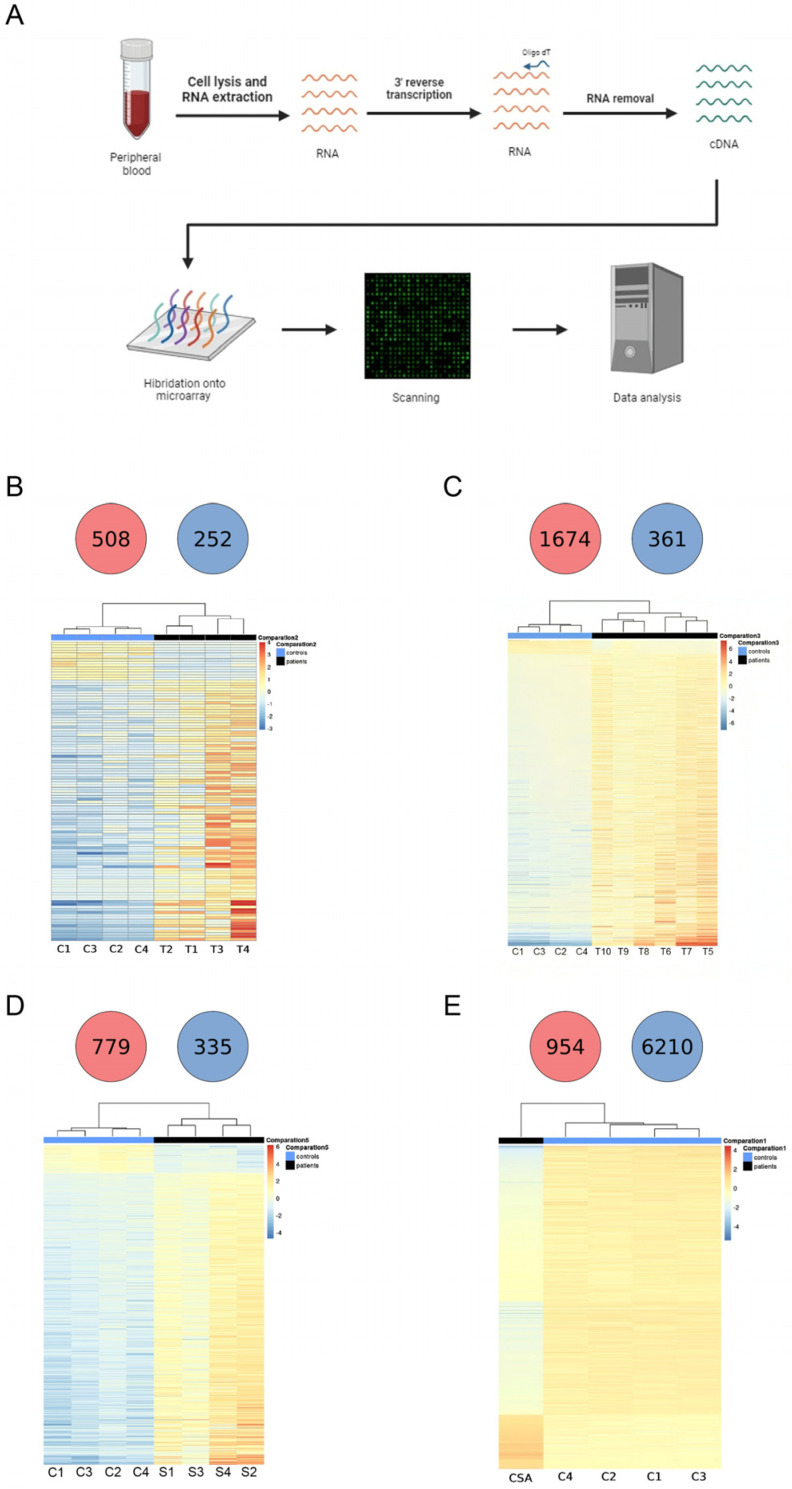
QuantSeq analysis in different congenital anemias. (**A**) General scheme of Quant 3′ mRNA–Sequencing procedure. (**B**–**E**) Heatmap and pie chart showing under- (blue) or overexpressed (red) genes between controls and transfusion-dependent thalassemia (TDT) (**B**), non-transfusion-dependent thalassemia (NTDT), (**C**), sickle cell disease (SCD), (**D**) and congenital sideroblastic anemia (CSA) patient. Heatmap is restricted to significant data according to Log2FC < −2 or >2, and adjusted *p*–value < 0.05.

The sequencing technique used in this study is called QuantSeq, which employs a simple and fast protocol to generate NGS libraries of sequences close to the 3′ end of polyadenylated RNAs. This technique’s advantage is that it produces only one fragment per transcript, making the number of reads mapped to a specific gene proportional to its expression (Figure 1A). Using DESeq2 software (https://www.bioconductor.org/packages/devel/bioc/html/DESeq2.html, accessed on 27 October 2024), a differential expression analysis was performed between the different groups under study and a control group consisting of four healthy individuals (two males and two females) with a median age of 40 years (29–53 years).

The dispersion of the samples indicates that the control individuals are very similar to each other, whereas the samples from TDT, NTDT, and SCD anemias show greater dispersion. In the case of the CSA sample, there is only one patient, but its dispersion shows a significant difference compared to the four healthy controls (Figure S1).

Transcriptome analysis identified 49,234 genes and compared their expression between groups. Accordingly, 508 upregulated genes and 252 downregulated genes were found in TDT patients compared to the controls (Figure 1B), 1674 upregulated genes and 361 downregulated genes in NTDT patients compared to the controls (Figure 1C), 779 upregulated genes and 335 downregulated genes in SCD patients compared to the controls (Figure 1D), and 954 upregulated genes and 6210 downregulated genes in the CSA patient compared to the controls (Figure 1E). To study the expression patterns in patient samples compared to control individuals, a restrictive threshold was established, considering only genes with a *p*-value < 0.05 and Log2FC < −2 or >2. Consequently, it can be observed that patients with TDT, NTDT, and SCD exhibit a higher number of upregulated genes compared to healthy controls (Figure 1B–D), whereas in the CSA patient, most genes are downregulated relative to healthy controls (Figure 1E).

### 2.2. The CSA Patient Exhibits a Distinct Expression Pattern in Genes Related to Erythropoiesis and Iron Metabolism, Glycolysis, Oxidative Metabolism, and Erythrocyte Membranes

**Figure 2:**
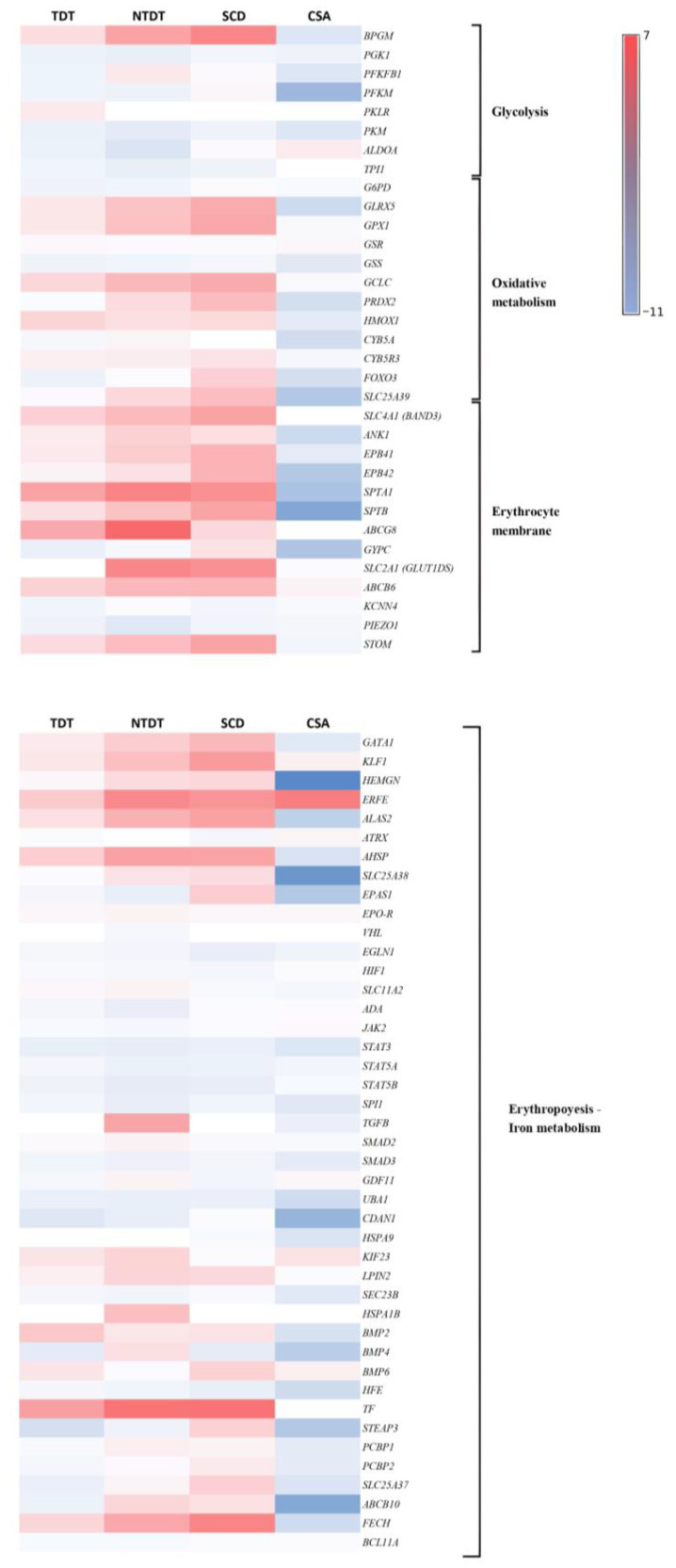
The CSA patient exhibits a distinct expression pattern in genes with respect to the rest of the congenital anemias. Heatmaps showing genes, which are significantly underexpressed (blue) or overexpressed (red) across different patient groups. The intensity of the colors indicates the level of expression of each representative gene. Groups studied correlate with those indicated in Figure 1 and pathways have been selected considering their importance in red blood cells, including erythropoiesis, iron metabolism, glycolysis, oxidative metabolism, and genes that codified erythrocyte membrane proteins.

To compare gene expression patterns across different types of congenital anemias, a comparative analysis was performed by grouping genes into various metabolic pathways known to be implicated in the development and progression of congenital anemias. The results indicate that the majority of the patients showed upregulation of the expression of genes related to erythropoiesis and iron metabolism, glycolysis, oxidative metabolism, and erythrocyte membranes compared to healthy donors. Surprisingly, the sideroblastic anemia patient (CSA) exhibited a distinct expression pattern in the pathways of erythropoiesis and iron metabolism, glycolysis, oxidative metabolism, and erythrocyte membranes, compared with the other anemias studied (Figure 2).

**Figure 3:**
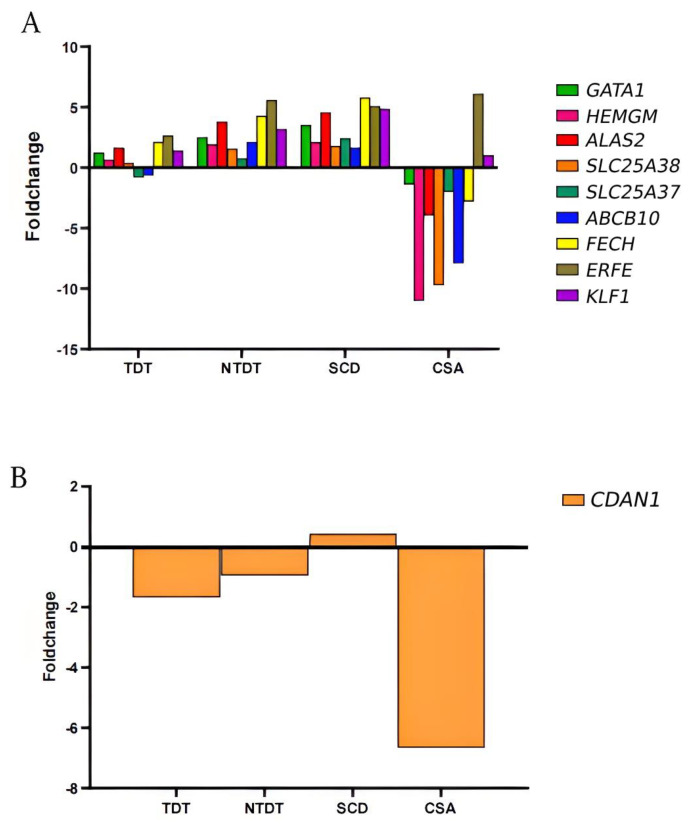
The CSA patient presents a different expression pattern of genes involved in erythropoiesis and iron metabolism. (**A**) Analysis of differential expressions of genes involved in erythropoiesis and heme group formation. Increase levels of *GATA1*, *HEMGN*, *ALAS2*, *SLC25A38*, *SLC25A37*, *ABCB10*, and *FECH* in all congenital anemias with respect to healthy control except the CSA patient. The increase in *ERFE* levels in all congenital anemias studied. (**B**) The *CDAN1* gene expression is significantly decreased in CSA patients. All graphs are represented in fold change with respect to control samples.

In particular, differences were observed in the expression pattern of the gene *GATA1*, the main erythroid transcription factor essential in early erythroid differentiation at the CFU-E and BFU-E levels [7]. In the cases of TDT, NTDT, and SCD patients, *GATA1* levels were elevated compared to controls, whereas in the CSA patient, the gene levels were reduced (Figure 3A). A similar pattern was observed for *HEMGN* (Hemogen), a transcriptional regulator involved in erythroid differentiation and proliferation in the bone marrow, whose function is related to the translocation of GATA1 to the nucleus [8]. Consistently, the *ALAS-2* gene, crucial for heme group synthesis and transcriptionally regulated by GATA1 [9], shows a similar expression pattern, being upregulated in TDT, NTDT, and SCD patients and downregulated in the CSA patient (Figure 3A).

*SLC25A38* is a gene that codifies mitochondrial glycine transport in erythroid progenitor cells necessary for the synthesis of heme (the prosthetic group of hemoglobin) and an essential substrate of ALAS2. As expected, this gene was downregulated only in CSA patients due to the disease being caused by an inherited mutation in this gene (Figure 3A). During erythroid differentiation, erythroid progenitor cells need to synthesize a large amount of heme for hemoglobin production, so proper expression of *SLC25A38* is essential [9].

Moreover, *SLC25A37* (mitoferrin-1), which is an essential gene for iron import into mitochondria that plays a crucial role in erythroid differentiation, was also downregulated in CSA. Mitoferrin-1 forms a complex with the protein ABCB10, an ATP-binding cassette transporter mitochondrial, and the enzyme ferrochelatase in the inner membrane of the mitochondria, and it has been reported that the expression of this protein is highly induced by the transcription factor GATA1 during erythroid differentiation [9]. Once again, our study pointed out a downregulation of this essential mitochondrial transporter that can be induced by low levels of GATA1, which were also downregulated in CSA (Figure 3A).

In addition, the gene *FECH*, a key ferrochelatase in heme group synthesis, *ABCB10,* and *KLF1* (which encodes a hematopoietic-specific transcription factor), showed the same expression pattern as genes discussed above (Figure 3A).

As expected, all the patients had a significant increase in the expression of the erythroferrone gene (*ERFE*), which could be explained by the state of hypoxemia secondary to chronic anemia presented in these patients (Figure 3).

The expression of *CDAN1* gene (encoding Codanin-1) was also analyzed because it is important in erythropoiesis and, in our study, only the CSA patient presented a significant decrease in *CDAN1* gene expression (fold change −6.6, *p* < 0.01). No significant differences were found in the rest of the study groups (Figure 3B).

### 2.3. BPGM Expression Is Particularly Upregulated in Homozygous SCD Patients

**Figure 4:**
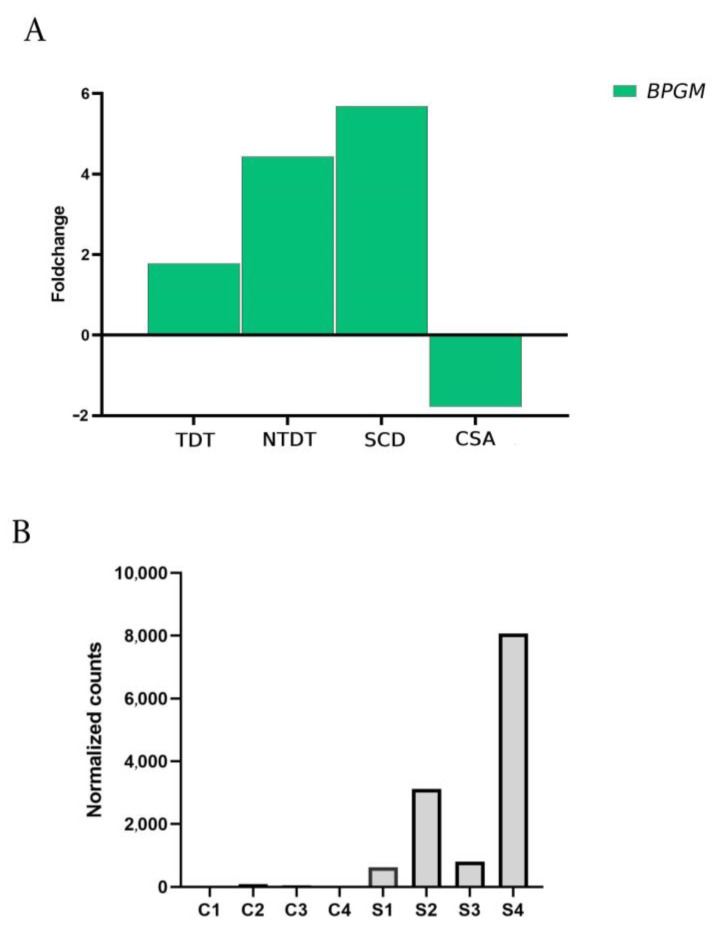
BPGM gene expression correlates with SCD patients’ severity. (**A**) Graph with the representative fold change expression compared to healthy controls for the *BPGM* gene in TDT, NTDT, SCD, and CSA patients. (**B**) *BPGM* normalized gene expression in the 4 healthy controls and the 4 SCD patients, with a clear increase in *BPGM* gene expression in S2 and S4 patients that correlates with patients’ disease severity.

Erythrocytes rely on the Embden–Meyerhof–Parnas (EMP) pathway, or glycolysis, for glucose metabolism [10,11]. During this process, 2,3-bisphosphoglycerate (2,3-BPG) is produced by bisphosphoglycerate mutase (BPGM). 2,3-BPG facilitates oxygen delivery to tissues, with its levels being closely associated with oxygen transport [11,12]. In our study, a significant increase in the expression of *BPGM* was observed in thalassemic and especially in SCD patients. These results suggest that this is a physiological increase in expression that attempts to compensate for hypoxemia secondary to a chronic hemolytic state [13]. However, in the case of the CSA patient, the expression is significantly reduced compared to the control group and the rest of the groups under study (Figure 4A).

In SCD patients, 2,3-BPG is involved in both the transfer of oxygen to tissues and the polymerization of sickle cell hemoglobin [12]. Therefore, the expression of *BPGM* was analyzed in each SCD patient to identify potential phenotype-dependent differences in expression. The highest expression levels were observed in patients homozygous for HbS (patients S2 and S4), correlating with a more severe clinical phenotype despite their younger age (Figure 4B).

### 2.4. The Majority of Congenital Anemias Show a High Expression Gene Profile Related to Oxidative Metabolism Compared to Healthy Donors

**Figure 5:**
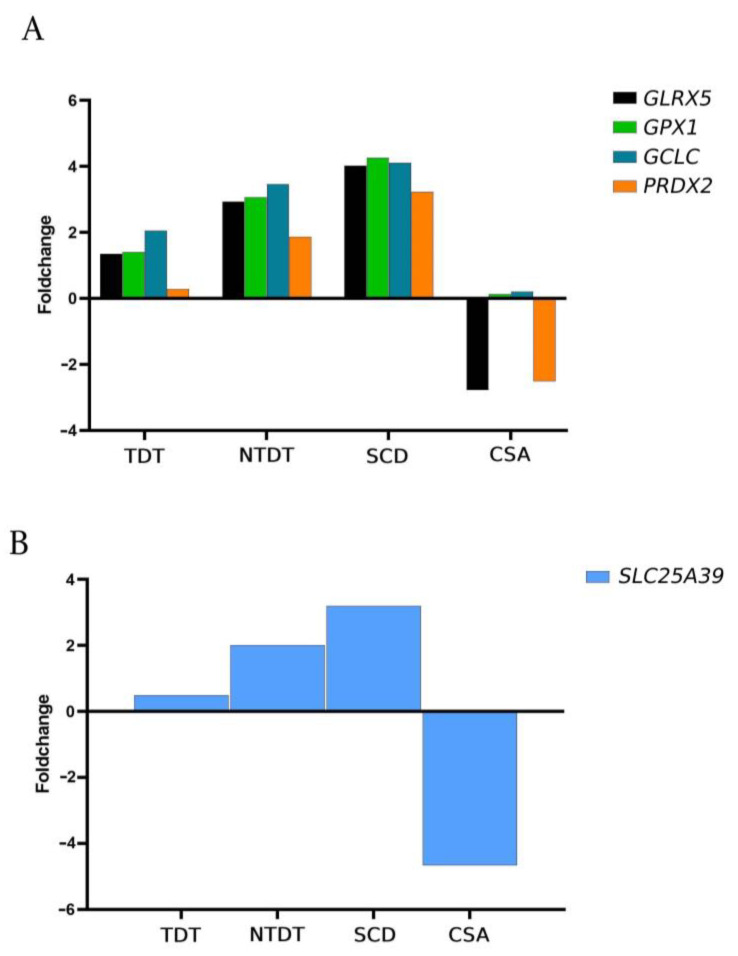
A high expression gene profile related to oxidative metabolism in the majority of the congenital anemias compared to healthy donors. (**A**) Genes related to oxidative metabolism, *GLRX5, GPX1, GCLC*, and *PRDX2*, are upregulated in all congenital anemias with respect to healthy controls except in the CSA patient. (**B**) Significant fold change decrease in *SLC25A39* gene expression in the CSA patient. All graphs represent the fold change relative to healthy control individuals.

The polymerization of sickle hemoglobin in SCD patients and the accumulation of unconjugated globin chains in thalassemia patients result in pathophysiological changes in erythrocytes and the endothelium, leading to severe oxidative stress causing erythrocyte rigidity, loss of membrane integrity, and hemolysis [14,15,16]. Therefore, the role of antioxidant enzymes is crucial in reducing the early lysis of hematocytes. In our study, we compared the expression of *GLRX5, GPX1*, and *GCLC* (implicated in glutathione metabolism) and *PRDX2* (involved in reactive oxygen species (ROS) metabolism), which are key enzymes in managing oxidative stress and iron homeostasis. Interestingly, in the TDT, NTDT, and SCD groups, these enzymes were overexpressed with respect to healthy donors, whereas in the CSA patient, *GLRX5* and *PRDX2* were downregulated, and *GPX1* and *GCLC* showed no significant differences compared to healthy controls (Figure 5A).

Given the crucial role of glutathione in oxidative metabolism, the expression of *SLC25A39* was examined across different groups of congenital anemias. Glutathione is produced in the cytosol and is primarily transported into mitochondria via the SLC25A39 carrier [17]. The results were consistent with those from oxidative metabolism gene analyses, showing increased expression in thalassemic and SCD patients, and decreased expression in the CSA patient compared to healthy controls (Figure 5B).

### 2.5. Significant Differences in Genes Related to Structural Membrane Protein Expression Between the Different Congenital Anemias

Patients with congenital anemias often exhibit an imbalance in the membrane and structural proteins of erythrocytes. Specifically, in thalassemia, globin chain imbalance disrupts the normal assembly of erythroid membrane proteins and erythroid precursors, contributing to the intramedullary lysis observed [18]. In SCD, the mutation in the hemoglobin leads to the formation of HbS, affecting the integrity of the erythrocyte membrane and cytoskeleton, increasing the rigidity of red blood cells, and promoting hemolysis [19].

**Figure 6:**
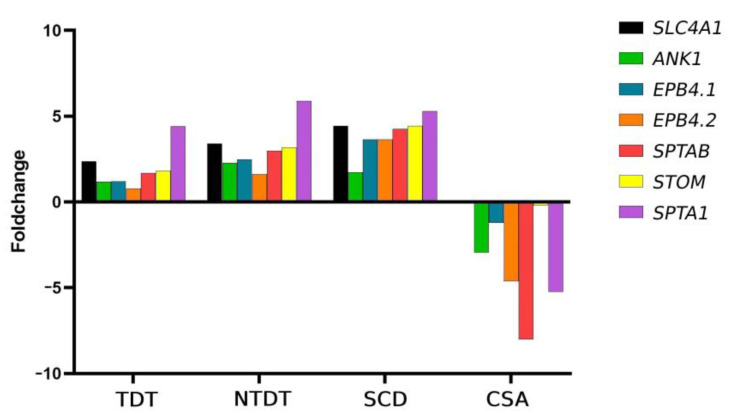
The genes related to structural membrane protein expression are differentially expressed in the different congenital anemias. *SCL4A1, ANK1, EPB4.1, EPB4.2, SPTB, STOM* and *SPTA1* gene expression profiles are represented for TDT, NTDT, SCD, and CSA patients. A decrease in expression in all of these genes is observed in the CSA patient. All fold changes are relative to healthy control individuals.

In our study, we found overexpression of the genes *SLC4A1*, *ANK1*, *EPB41*, *EPB42*, *SPTA1*, *SPTB*, and *STOM*, all of which are involved in maintaining erythrocyte structure and function, in patients with thalassemia and SCD. However, in the case of the CSA patient, once again, these genes were clearly downregulated compared to healthy controls (Figure 6).

### 2.6. Other Pathways Altered in Each Congenital Anemia

**Figure 7:**
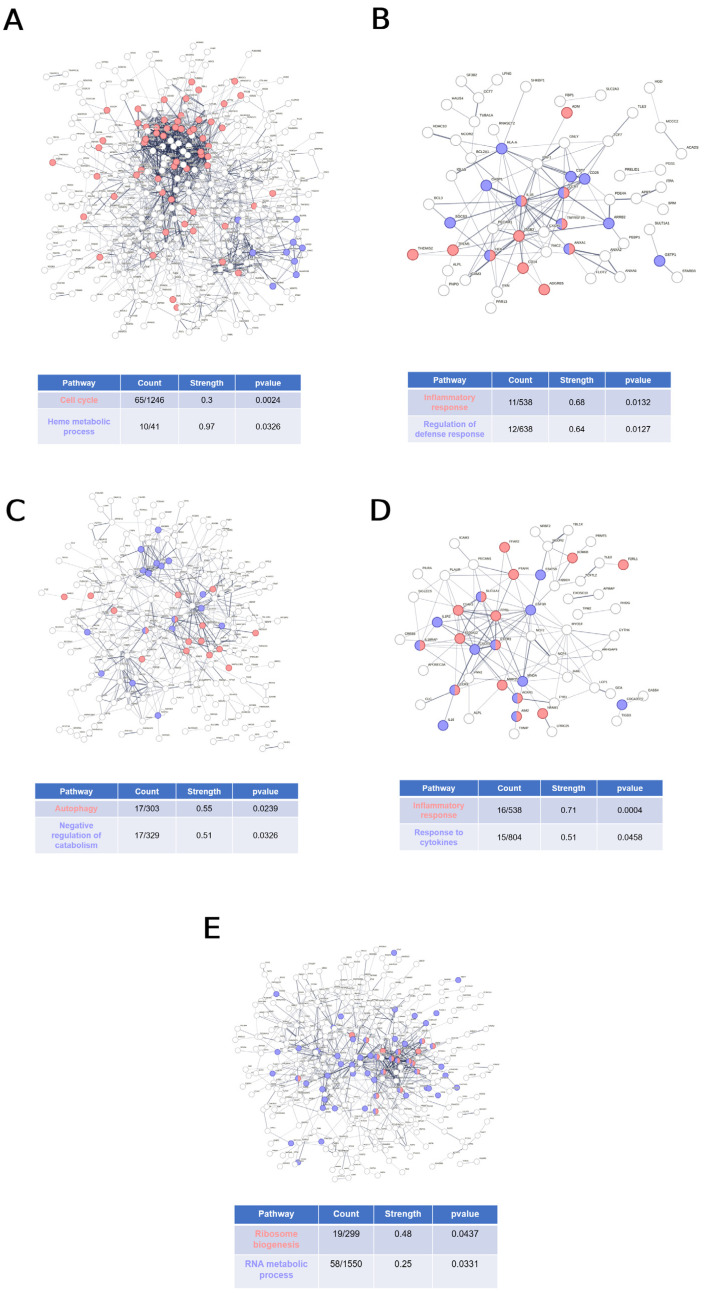
Dysregulated metabolic pathways in patients with congenital anemias (**A**–**E**). STRING analysis of interactions among upregulated genes in NTDT (**A**) and SCD (**C**) patients, and downregulated genes in NTDT (**B**), SCD (**D**), and CSA (**E**), compared to healthy controls. The table highlights metabolic pathways significantly impacted, accounting for the total number of genes involved in each network, their strength (Log10(genes implicated/total number of genes in the network)), and significance (*p*-values corrected for multiple testing within each category using the Benjamini–Hochberg procedure).

In addition to the study of the canonical pathways involved in the development of congenital anemias, an analysis of the metabolic pathways affected in each group of patients compared to healthy individuals was conducted using STRING. This analysis employed a more stringent selection criteria, considering only the genes with a *p*-value < 0.01 and Log2FC < −2 or >2. The results indicated that TDT patients did not exhibit any significantly altered metabolic pathways other than those previously discussed, which were related to oxygen transport. In contrast, NTDT patients presented upregulated genes associated with the cell cycle (Figure 7A) and downregulated genes involved in the inflammatory response and defense response regulation (Figure 7B). Conversely, SCD patients exhibited upregulated genes involved in autophagy and the negative regulation of catabolism (Figure 7C). Interestingly, the downregulated genes in these patients were involved in the inflammatory and cytokine response (Figure 7D), like NTDT patients (Figure 7B). Finally, the CSA patient presented upregulated genes that did not significantly relate to any pathway and downregulated genes associated with ribosome biogenesis and RNA metabolic processes (Figure 7E).

## 3. Discussion

Erythropoiesis is a complex and dynamic process involving numerous metabolic pathways. In many congenital anemias, a simple genetic alteration is the driver of the primary cause of the disease. However, this primary alteration often leads to multiple secondary processes that are altered and deregulated. Our study delves into the RNA expression levels of these processes to understand their involvement in different forms of congenital anemias. We observed clear differential expression patterns between patients with congenital hemolytic anemia (thalassemia and SCD) and congenital anemia due to bone marrow failure (CSA with the *SLC25A38* mutation) and then compared them to healthy controls [1].

In these diseases, due to ineffective erythropoiesis, the final production of erythrocytes is insufficient, not only in quantity but also in quality. Thus, circulating erythrocytes may show deficiencies in their cellular components and metabolic systems such as the composition of the cell membrane, the enzymatic systems that participate in energy and oxidative metabolism, etc., that all contribute to a decrease in survival. Furthermore, there is an antiapoptotic mechanism of HbF during the terminal stages of erythroid differentiation that positively selects cells with high levels of fetal hemoglobin (HbF) and contributes to a decrease in ineffective erythropoiesis [20,21].

In our study, we observed increased expression of *GATA1* (the master regulator of erythropoiesis) and other genes involved in erythroid differentiation in patients with thalassemia and SCD. This could be due to a compensatory mechanism for ineffective erythropoiesis and chronic hemolysis, as has been described in patients with SCD outside of VOC [22].

Moreover, a significant increase in the expression of genes involved in erythroid differentiation and proliferation such as *KLF1*, and *HEMGN*, and the formation of the HEMO group (*ALAS2*, *SLC25A38*, and *FECH*) was observed in thalassemic and SCD patients. In addition, a correlation was observed between the expression of *GATA1*, *SLC25A37*, and *ABCB10* in the transfusion-independent group. These results could be explained by the increased erythroid proliferation in the context of the ineffective erythropoiesis that occurs in these patients. However, in the patient with CSA, the expression is again decreased. This could be explained by the fact that the patient presents an alteration in the synthesis of the HEMO group.

Moreover, in the case of the CSA patient, we see a global decrease in expression in genes involved in erythropoiesis. This has been described in myelodysplastic syndrome (MDS) patients, where a low expression of *HEMGN* contributes to intranuclear delocalization of GATA1 and may contribute to the worsening of ineffective erythropoiesis [8]. In the case of our patient with CSA-SCL25A38, we observed a significant decrease in this gene, which leads us to believe that the mechanism by which the dyserythropoiesis observed in patients with anemias due to bone marrow failure occurs is different from that observed in other congenital hemolytic anemias.

Also, what is striking is the low expression of *CDAN1*, a gene involved in congenital dyserythropoietic anemia type I (CDA-I) [23]. Its function is not very well known, but it is believed to regulate the incorporation of histones into DNA during cell replication [23]. Moreover, mutations in this gene cause congenital dyserythropoietic anemia type I [24]. In our study, only the CSA patient had a significantly decreased expression of this gene. Thus, the low expression found in the *CDAN1* gene could contribute to altered erythroid maturation in patients with other types of congenital anemia due to bone marrow failure.

In addition, increased glycolytic enzymes have been reported in patients with SCD, which is attributed to an increased demand for ATP synthesis. Published proteomic data suggest increased levels of the enzyme 2,3-bisphosphoglycerate kinase, supporting the theory of stimulation of glycolytic pathways in SCD [25]. Our data support the idea that genes involved in anaerobic erythrocyte metabolism show increased expression in SCD patients, although not significantly. Again, the difference with respect to the expression in the patient with CSA, in whom the expression of these glycolytic genes is globally decreased, is surprising.

In this context, significantly elevated concentrations of 2,3-BPG have been described in sickle erythrocytes as an adaptive response secondary to chronic hemolytic anemia. However, this response is counterproductive as it leads to an increase in deoxygenated Hb by promoting polymerization of the sickle erythrocyte [12]. Thus, published data suggest that 2,3-BPG levels are directly involved in the stabilization of HbS polymers [26]. Its decrease in sickle cells has been shown to reduce HbS polymerization. In our study, the data obtained in SCD patients support those published in the literature. We have also found a significant increase in patients with SCD. However, in the case of the patient with CSA, the expression is significantly reduced with respect to the control group and the rest of the study groups.

On the other hand, it has been described that in thalassemic patients and patients with SCD, there is an increase in oxidative stress secondary to free iron and hemoglobin, due to the disbalance of chains in the case of thalassemic patients and to repeated VOC in patients with SCD [14,27,28]. Increased expression of genes with antioxidant activity seems to be common in most studies carried out in patients with SCD, especially the increase in catalase and peroxiredoxins, in the context of a compensatory increase in antioxidant mechanisms in this context of a high oxidative load in SCD [29].

Our results showed that pathways involved in the response to oxidative stress (*GLRX5*, *GPX*, *GCLC*, and *PRDX2*) were more activated in thalassemic patients and SCD patients compared to healthy individuals, corroborating the findings published in the literature. However, the behavior in the CSA patient is the opposite, showing significantly reduced expression levels with respect to controls and other congenital anemia groups.

In addition, mitochondrial glutathione is required for the activity and stability of proteins containing iron and sulfur groups, as well as for erythropoiesis and oxidative metabolism [17]. In our study, we observed a significant decrease in the expression of the *SLC25A39* gene coding for the mitochondrial glutathione transporter, which was not observed in the other groups under study. Our hypothesis is that the SLC25A39 transporter could play a role in the pathophysiology of CSA.

Related to membrane structural proteins, it has been reported by proteomic studies that sickle cell erythrocytes show trends in the upregulation of cytoskeletal defects. Many of these findings are consistent with the pathophysiology of sickle cell disease, including high oxidative burden, resulting in damage to cytoskeletal and other proteins and erythrocyte rigidity [25]. In addition, anaerobic glycolysis depends on the expression of certain membrane proteins [12], which we find overexpressed in thalassemia and SCD. In our analysis, we observed a global increase in genes coding for structural membrane proteins in the groups with congenital hemolytic anemia (SCD and thalassemia), while this premise was not met in the patient with anemia due to bone marrow failure.

Finally, other important pathways like the cell cycle, inflammatory and defense responses, or autophagy seem to be modulated differently in the studied anemias. Therefore, this study opens new avenues to deeply explore these rare diseases.

All in all, our study provides a new map of gene expression profiles including different types of congenital anemias that could be used as new biomarkers in the clinic. Our findings reveal a distinct differential expression profile in the SLC25A38 patient compared to other congenital anemia groups. Despite the limited number of CSA patients in our study, due to the rarity of this type of disease and its infrequent etiology, this research provides critical insights into the transcriptomic profiles of congenital anemias and highlights the need for further investigation to understand and predict biomarkers in congenital anemias.

## 4. Materials and Methods

### 4.1. Study Design and Patient Cohort

A whole blood differential expression analysis was performed on 15 patients undergoing follow-up in the erythropathology department of the Hospital Clínico Universitario Virgen de la Arrixaca (HCUVA) with a diagnosis of congenital anemia. Of the 15 patients studied, 4 (27%) were diagnosed with TDT, 6 (40%) with TNTD, 4 (27%) with SCD, and 1 (6%) with CSA. The patient included with PKD deficiency also had heterozygous β-thalassemia, so to facilitate the analysis, she was included in patients with TTD. The clinical characteristics of the patients are presented in Supplementary Tables S1–S3.

### 4.2. Blood Samples Collection

Patient samples were provided by the Hematology Service at HCUVA with ethics approval number (2021-11-9-HCUVA; 2023-3-5-HCUVA). Blood samples were collected from 4 healthy donor controls, 4 TD thalassemic patients, 6 NTD thalassemic patients, 4 SCD patients, and 1 SLC25A38 CSA patient.

### 4.3. RNA Extraction and Sequencing

RNA was extracted from whole blood samples using a NucleoSpin RNA blood mid kit (Macherey-Nagel, Düren, NRW, Germany). Subsequently, the RNA library was built using the QuantSeq 3′ mRNA-Seq Library Prep Kit FWD (Lexogen, Vienna, Austria). Before sequencing, RNA concentration was measured using the QIAxcel Advanced System device (Qiagen, Hilden, Germany) to verify the quality of the library. Finally, all samples were sequenced following the Illumina protocol for the Hiseq XTen (150 × 2) device. Raw data were obtained in a FASTQ format and used for bioinformatic analysis (Figure 1A).

### 4.4. Quality Control, Trimming, and Alignment to the Reference Genome

The selection of the high-quality sequences from raw data (trimming) was made by the pipeline, as well as the elimination of the adapters from the sequences. After this process, the FastQC^2^ tool was used to study the quality of the final FASTQ files. Samples must have some features such as an acceptable % Q20, lack of adaptors, lack of Ns, poliA sequences, etc. The reads generated in sequencing were aligned to the reference genome using the STAR^3^ software (https://www.encodeproject.org/software/star/, accessed on 27 October 2024). This software uses the alternative-splicing database in the alignment of the reads.

### 4.5. Analysis of Differential Expression

HTSeq-count software (https://htseq.readthedocs.io/en/latest/, accessed on 27 October 2024) was used to count the reads of each gen using *.gtf files from NCBI. The higher the number of reads of one gene was, the higher its expression correlated. However, to compare expressions between genes, it was necessary to normalize the database considering different factors. In this case, the information obtained was processed using the DESeq2^5^ pipeline.

PCA (principal component analysis) was used to evaluate the similarity between samples based on the normalized counts to estimate the quality of the experiment. The similarity of the expression in samples was used to cluster the information by the disease of each group with respect to healthy donors.

To process the data for easy visualization, different software from Bioconductor^®^ software (https://www.bioconductor.org/, accessed on 27 October 2024) was used (DESeq2, ggplot, pheatmap, stringr, etc.).

Data were collected and represented in grouped bar graphs using GraphPad Prism 9. These graphs show the upregulation or downregulation of different genes based on log 2 FC values.

### 4.6. String Analysis

The String database (Search Tool for the Retrieval of Interacting Genes/Proteins) integrates the known information on protein–gene interactions. This database combines experimental data, computational prediction results, and analysis of the published literature. The data are structured as a network, where the nodes represent genes or proteins, and the links represent physical or functional interactions. Each of these interactions has a “*p*-value” based on scientific evidence (https://cn.string-db.org/cgi/about, accessed on 27 October 2024).

## 5. Conclusions

We have identified that the pathogenic alteration of SLC25A38 in a CSA patient is accompanied by significant and distinct changes in the differential expression of genes involved in essential processes of erythroid differentiation. Notably, there is a low expression of various genes related to oxidative stress (*GLRX5*, *GPX*, *GCLC*, and *PRDX2*), with a significant decrease in *SLC25A39* expression potentially contributing to a more severe disease phenotype. Additionally, we observed a global increase in the expression of glycolytic genes, with a significant expression pattern of *BPGM* in SCD patients that correlates with disease severity. Other relevant findings include the low overall expression of membrane genes and genes involved in erythropoiesis (*GATA1*, *ALAS2*, *ABCB10*, and *HEMGN*) in CSA and overexpression in the rest of the congenital anemias studied compared to healthy controls. These alterations together may contribute to the worst phenotype of CSA, or they may be a secondary consequence of the primary SLC25A38 alterations. These findings provide insights into the distinct behaviors of hereditary hemolytic anemias, wherein ineffective erythropoiesis predominates, whereas anemias due to bone marrow failure are characterized by altered oxidative stress mechanisms.

## Data Availability

The data that support the findings of this study are available upon request from the corresponding author.

## Supplementary Materials

The following supporting information can be downloaded at https://www.mdpi.com/article/10.3390/ijms252111706/s1.
