# Supplementary material for: Whole Blood Transcriptome Analysis in Congenital Anemia Patients
Source: Int J Mol Sci. 2024 Oct 31;25(21):11706. doi: 10.3390/ijms252111706 (PMC11546763; doi:10.3390/ijms252111706)
Supplement: Supplementary file 1 [file ijms-25-11706-s001.zip › ijms-3262030-supplementary.pptx]

## Slide 1
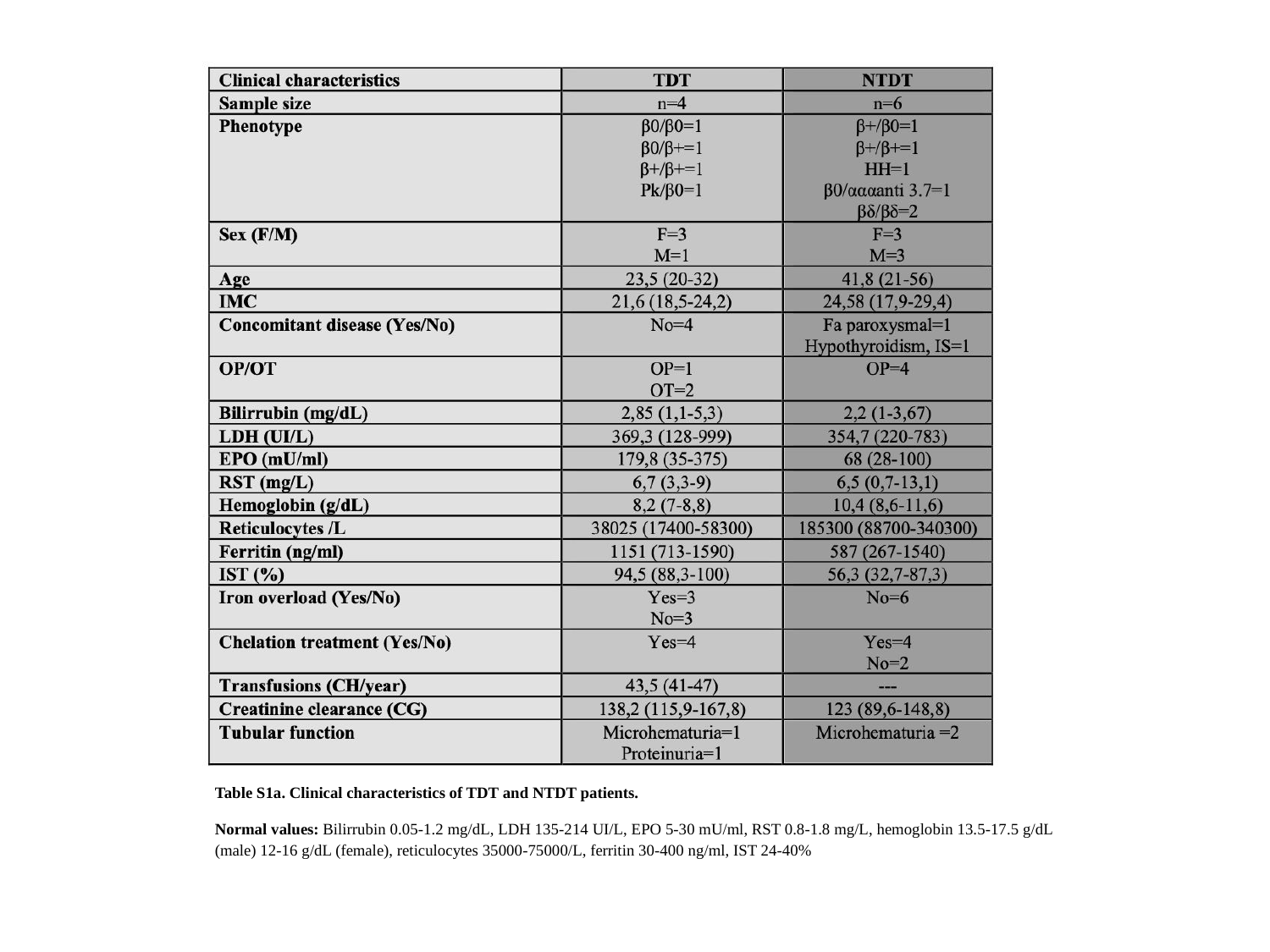

Table S1a. Clinical characteristics of TDT and NTDT patients.
Normal values: Bilirrubin 0.05-1.2 mg/dL, LDH 135-214 UI/L, EPO 5-30 mU/ml, RST 0.8-1.8 mg/L, hemoglobin 13.5-17.5 g/dL (male) 12-16 g/dL (female), reticulocytes 35000-75000/L, ferritin 30-400 ng/ml, IST 24-40%

## Slide 2
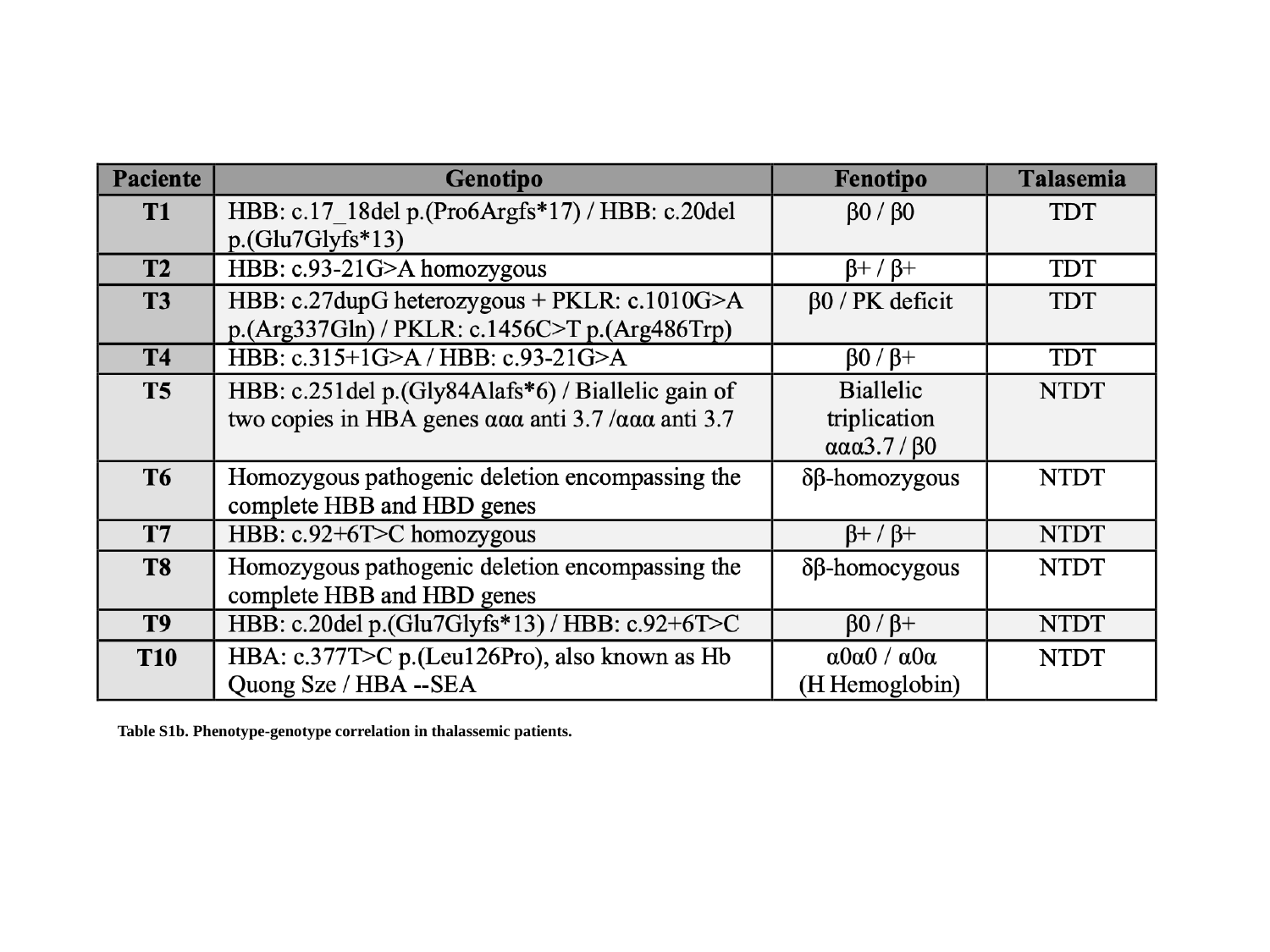

Table S1b. Phenotype-genotype correlation in thalassemic patients.

## Slide 3
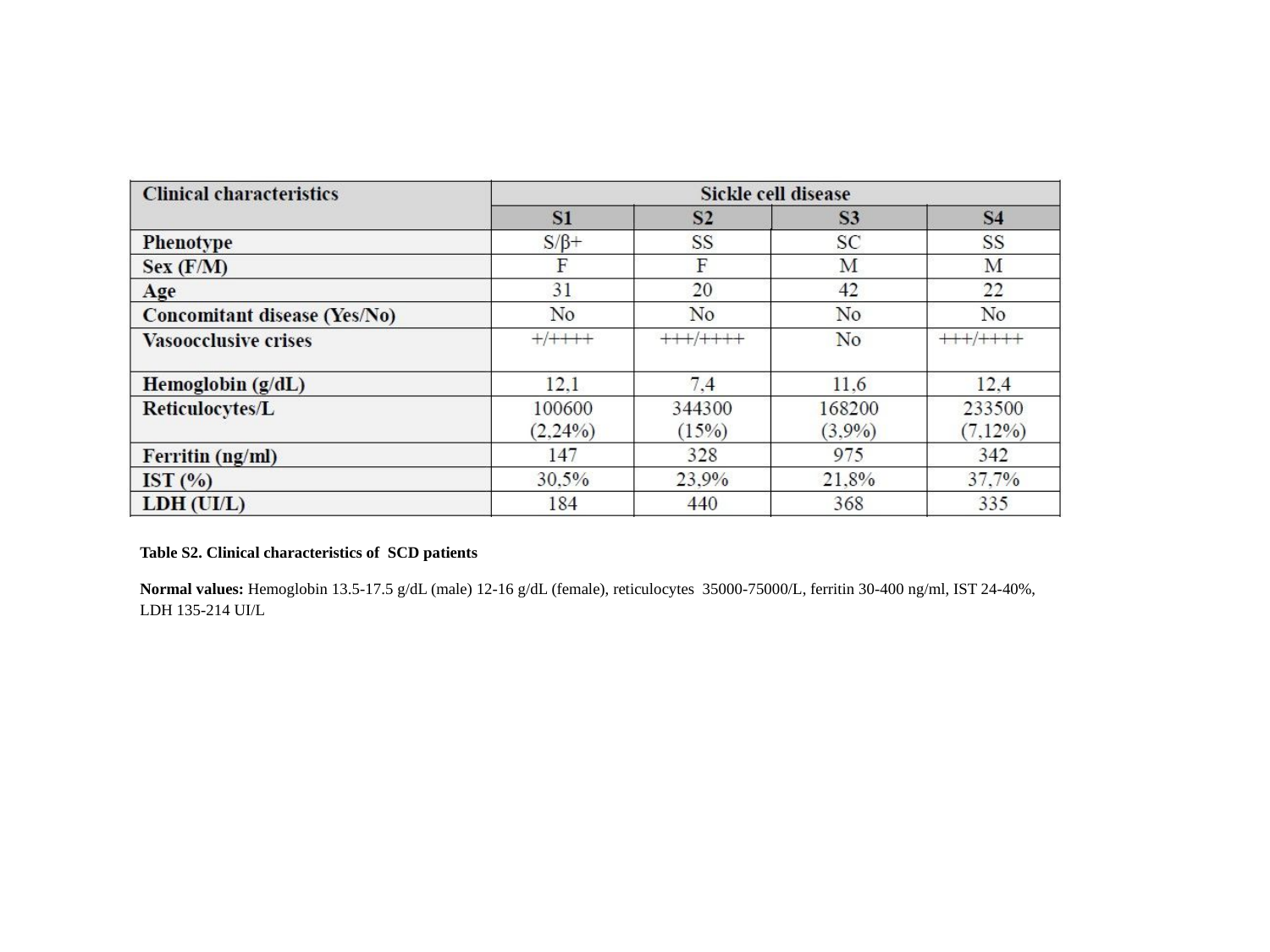

Table S2. Clinical characteristics of SCD patients
Normal values: Hemoglobin 13.5-17.5 g/dL (male) 12-16 g/dL (female), reticulocytes 35000-75000/L, ferritin 30-400 ng/ml, IST 24-40%, LDH 135-214 UI/L

## Slide 4
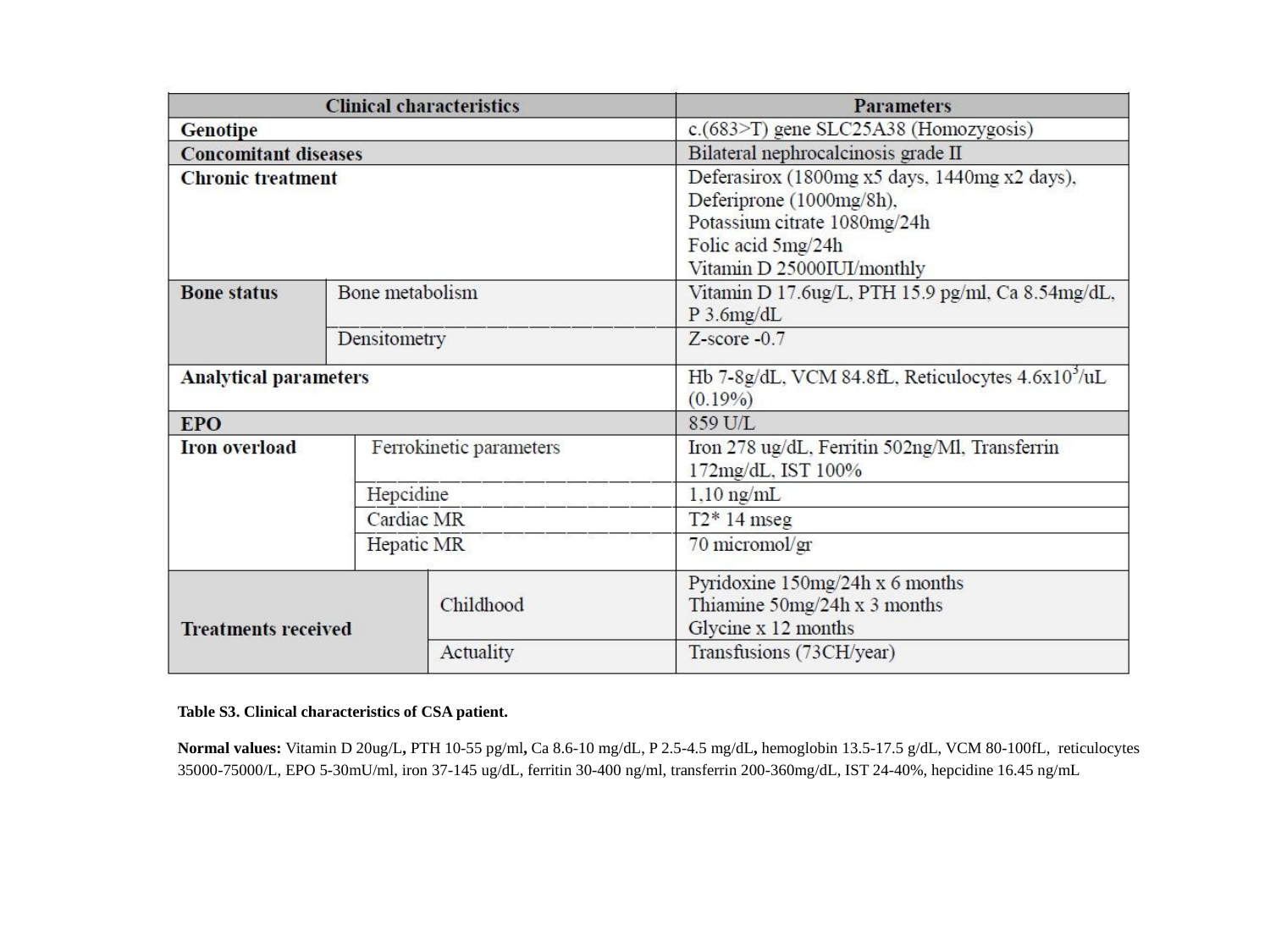

Table S3. Clinical characteristics of CSA patient.
Normal values: Vitamin D 20ug/L, PTH 10-55 pg/ml, Ca 8.6-10 mg/dL, P 2.5-4.5 mg/dL, hemoglobin 13.5-17.5 g/dL, VCM 80-100fL, reticulocytes 35000-75000/L, EPO 5-30mU/ml, iron 37-145 ug/dL, ferritin 30-400 ng/ml, transferrin 200-360mg/dL, IST 24-40%, hepcidine 16.45 ng/mL

## Slide 5
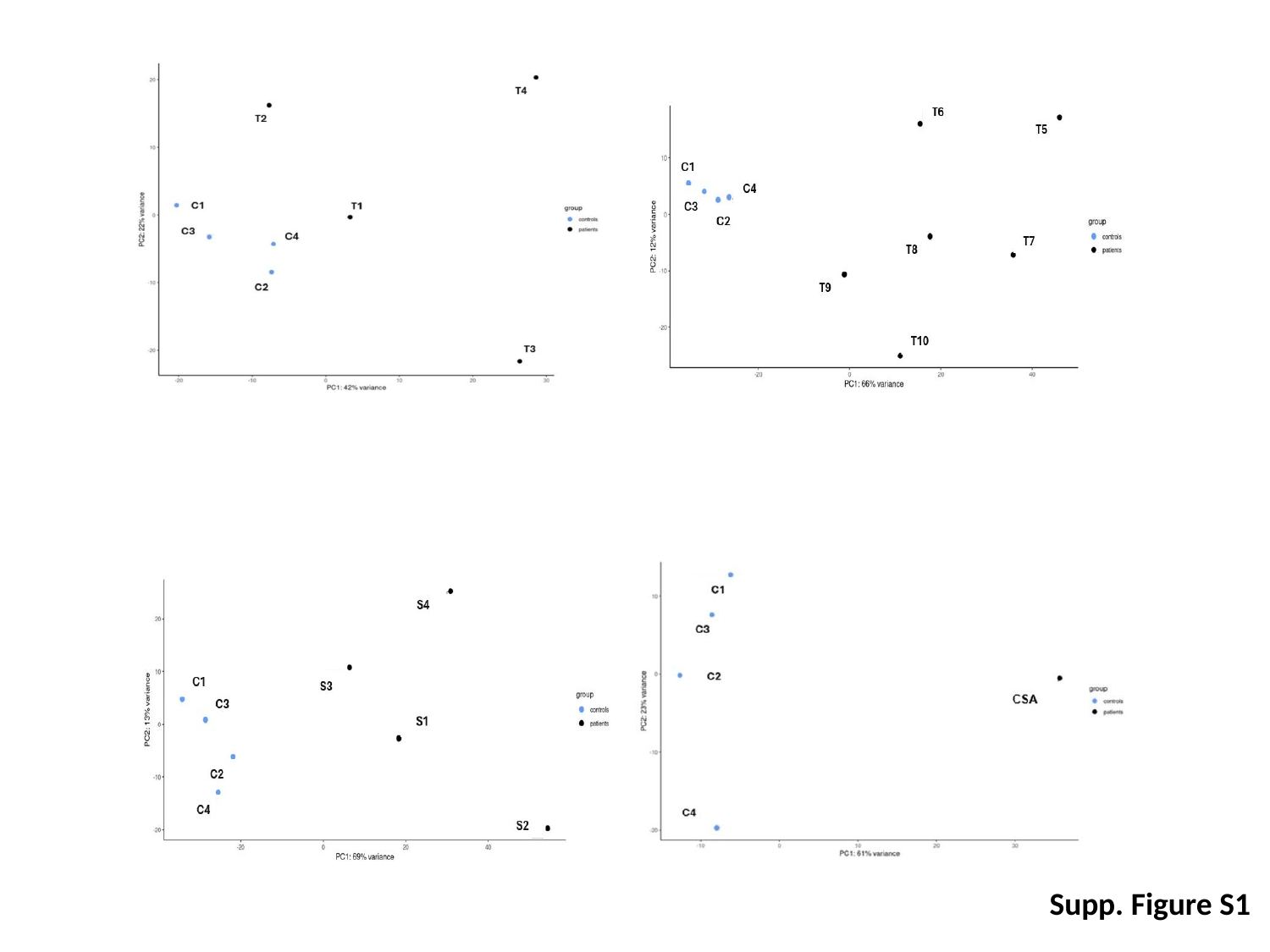

Supp. Figure S1

## Slide 6
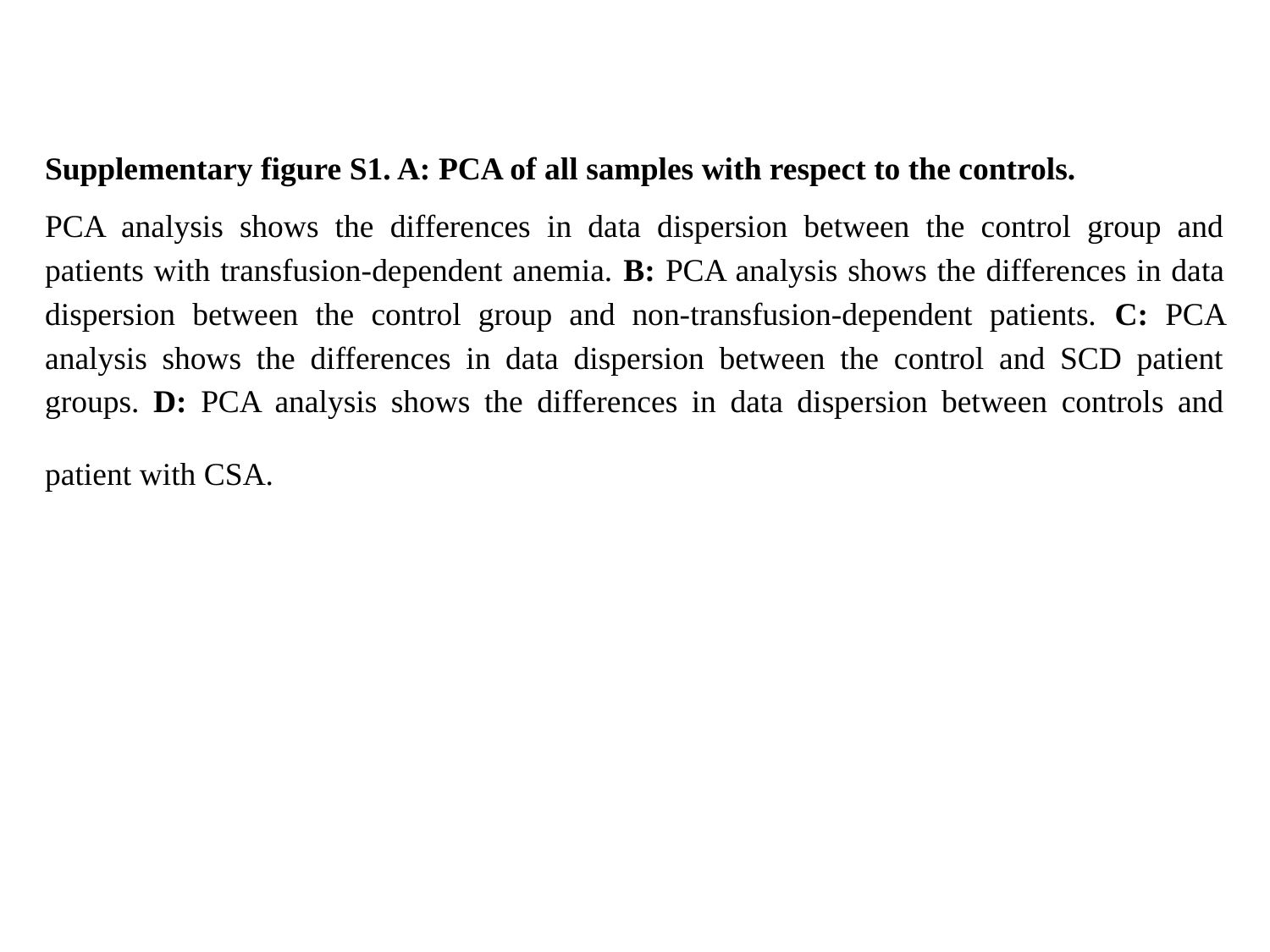

Supplementary figure S1. A: PCA of all samples with respect to the controls.
PCA analysis shows the differences in data dispersion between the control group and patients with transfusion-dependent anemia. B: PCA analysis shows the differences in data dispersion between the control group and non-transfusion-dependent patients. C: PCA analysis shows the differences in data dispersion between the control and SCD patient groups. D: PCA analysis shows the differences in data dispersion between controls and patient with CSA.
